# 

**Published:** 1889-02

**Authors:** 


### SPECIAL.

We desire to say to such of our patrons as are in arrears, that we should esteem it a favor if they would promptly remit their dues for the present year. One is apt to reason with himself that his single dollar can be of little concern to us, but when these delinquencies are counted by thousands, they are seriously felt. Our endeavor is to give every subscriber the full value of his money by furnishing the very best family health journal published. If we fail in this it is not for want of interest and pains-taking. All we require is the right kind of encouragement.

Please remit promptly by postal order, and leave us your debtor for a kindly and considerate act.

### INDEX.

We have published a TITLE PAGE and INDEX to the Volume 1888, of this Journal, with which any subscriber will be furnished free, on application to this office, or to the American News Co., Chambers Street, N. Y. City.

### BOOK NOTICES.

### NEW EXCHANGES.

THE ALABAMA MEDICAL AND SURGICAL AGE.—Anniston. Monthly. $2.00 per annum.

THE PHARMACEUTICAL ERA.—Detroit, Mich. Monthly. $1.50 per annum.

THE SANITARY VOLUNTEER.—Concord, N. H. Monthly. 50 cents per annum.

THE TRAINED NURSE.—Buffalo. Monthly. $1.00 per annum.

PHILOSOPHY OF NATURE.—Monthly. 261 Broadway, New York City. $1.00 per annum.

NATIONAL LIBERATOR.—Boston. Monthly. $1.00 per annum.

Our thanks are due for the following publications :

THE SLÖJD IN THE SERVICE OF THE SCHOOLS.—New York Educational Association.

THE PULLEY METHOD IN SURGERY.—Prof. A. E. Prince, Jacksonville, Ill.

THE EXTRACTION OF CATARACT.—By the same.

CLUB FOOT, ETC.—David Prince, M. D., Jacksonville, Ill.

THE AMERICAN BIOGRAPHICAL PUBLISHING CO., Philadelphia, have in press a volume entitled "American Resorts," by Bushrod W. James, A. M. M. D., intended for invalids, etc., etc.

### BOOKS.

We are in receipt of copies of THE TRAINED NURSE, a monthly magazine consecrated to those who minister to the sick and suffering in hospital and home. It is published by the Lakeside Publishing Co., of Buffalo, N. Y., and its editor, Margaret Elliott Francis, was formerly Superintendent of the Buffalo Training School for Nurses.

THE CENTURY MAGAZINE for February is fully up to its own high standard of excellence. It has recently published an enlarged map of Siberia, showing the route taken by George Kennan, author of its interesting and instructive Siberian papers not yet concluded. The Century Co., New York City.

THE MANUFACTURER AND BUILDER.—Monthly. 33 Nassau street, New York City. Comes to us in a new and highly artistic dress. Its admirable illustrations and explanatory text of the more important inventions are unsurpassed. $1.50 per annum.

A DREAM BEYOND THE GRAVE.—By John Franklin Clark. This is a drama founded upon the spiritualistic philosophy of the after life, largely out of the manifold researches of the author in the course of earnest seeking from various sources of knowledge of super-mundane affairs. Sold by American News Co., N. Y.

WARNER'S VOICE MAGAZINE, changed from *The Voice*, a journal devoted to all phases of the human voice, organic and functional. Office 28 West Twenty-third st.

Messrs. J. B. Lippincott Company announce to the profession the publication of a CYCLOPÆDIA OF THE DISEASES OF CHILDREN, medical and surgical, by American, British and Canadian authors, edited by John M. Keating, M. D., in four imperial octavo volumes ; to be sold by subscription only. The first volume will be issued early in April and the subsequent volumes at short intervals.

A thorough knowledge of the diseases of children is a matter of the greatest importance to most physicians, and as this is the only work of the kind that has been published in English, it will be invaluable as a text-book and work of reference for the busy practitioner.

